# The effect of different cutoff schemes in molecular simulations of proteins

**DOI:** 10.1002/jcc.26426

**Published:** 2020-10-07

**Authors:** Matthias Diem, Chris Oostenbrink

**Affiliations:** ^1^ Institute for Molecular Modeling and Simulation University of Natural Resources and Life Sciences Vienna Austria

**Keywords:** atomistic, charge‐group, cutoff scheme, molecular dynamics, proteins

## Abstract

Molecular simulations of nanoscale systems invariably involve assumptions and approximations to describe the electrostatic interactions, which are long‐ranged in nature. One approach is the use of cutoff schemes with a reaction‐field contribution to account for the medium outside the cutoff scheme. Recent reports show that macroscopic properties may depend on the exact choice of cutoff schemes in modern day simulations. In this work, a systematic analysis of the effects of different cutoff schemes was performed using a set of 52 proteins. We find no statistically significant differences between using a twin‐range or a single‐range cutoff scheme. Applying the cutoff based on charge groups or based on atomic positions, does lead to significant differences, which is traced to the cutoff noise for energies and forces. While group‐based cutoff schemes show increased cutoff noise in the potential energy, applying an atomistic cutoff leads to artificial structure in the solvent at the cutoff distance. Carefully setting the temperature control, or using an atomistic cutoff for the solute and a group‐based cutoff for the solvent significantly reduces the effects of the cutoff noise, without introducing structure in the solvent. This study aims to deepen the understanding of the implications different cutoffs have on molecular dynamics simulations.

## Graphical abstract

Molecular dynamics simulations of nanoscale systems invariably involve assumptions and approximations to describe electrostatic interactions. In this study, we compare four different cutoff schemes on a set of 52 proteins. Statistically there are no significant differences between using a twin‐range or a single‐range cutoff scheme. Applying the cutoff based on charge groups or based on atomic positions does lead to significant differences, the sources and effects are discussed.

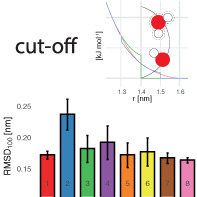

## 1 INTRODUCTION

Molecular dynamics simulations are an invaluable tool to study the behavior of proteins in aqueous solution in great detail. Nowadays time scales up to milliseconds can be simulated, which lead to new insights, that were not possible before.^[^ ^1^ ^]^ Prolonged simulations possibly bring to light new challenges in the development of reliable force fields as well as effects of assumptions and approximations in algorithms that have been widely used.^[^ ^2^, ^3^, ^4^ ^]^ The biggest part of computer time is used to identify and calculate the nonbonded interactions.

One way of treating the nonbonded interactions is based on lattice summation schemes. These methods make use of the commonly applied periodic boundary conditions and assume a periodic repetition of charges at an infinite range.^[^ ^4^, ^5^ ^]^ This assumption is challenged in systems, which are not perfectly periodic but should represent a dilute solution of biomolecules. While lattice summation methods are very commonly applied, these methods are not without artifacts. The induced periodicity leads to an underrepresentation of the electrostatic interactions (unlike charges at exactly half the box‐length have no interaction). This underpolarization has effects on the calculation of thermodynamic properties as well as the structures sampled in simulations. The effects on system properties have been described repeatedly.^[^ ^6^, ^7^, ^8^, ^9^, ^10^ ^]^

Another way to treat electrostatic and van der Waals interactions are cutoff schemes, in which interactions are only computed up to a fixed atomic or molecular distance. Since a straight truncation leads to major artifacts,^[^ ^11^, ^12^ ^]^ a reaction‐field contribution combined with shifting or switching functions are used to ensure that the energy approaches zero at the cutoff distance. For the reaction‐field contribution, a continuous medium outside the cutoff‐region is assumed.^[^ ^13^, ^14^ ^]^ Given a box size that is larger than twice the cutoff distance, these approaches do not show artifacts due to periodicity. However, the neglect of molecular detail beyond the cutoff distance does affect the thermodynamics of the system in different ways, in particular for charged species.^[^ ^7^, ^8^, ^9^ ^]^ A cutoff can be either imposed based on interatomic distances or by using charge‐groups. In the latter case, the molecular interactions between all atoms that are part of two charge‐groups interact as long as the centers of the charge‐groups are within the cutoff distance. The advantage of this approach is, that the definition of neutral charge groups reduces the vast majority of the electrostatic interactions to dipole–dipole interactions which have a shorter range than charge–charge interactions (*r* ^−3^ vs. *r* ^−1^). For efficiency reasons, the electrostatic interactions within an atomistic or charge‐group‐based cutoff scheme are typically calculated from a pairlist that is not necessarily updated at every timestep of the simulation. In addition, the GROMOS force fields that will be used in the current work were parameterized with a twin‐range cutoff scheme. In this approach, a pairlist is calculated at specific time intervals (e.g., every 10 fs). Short‐range interactions, for example, up to 0.8 nm are computed at every timestep from this pairlist. Upon pairlist construction, interactions up to a longer range cutoff (e.g., 1.4 nm) are also computed and kept constant between pairlist updates. The twin‐range cutoff scheme is a way to speed up simulations and allow for longer simulation timescales, but it also introduces discontinuities in the nonbonded energies and forces which leads to additional noise in the simulation. Therefore, it is crucial to fine‐tune the update intervals, an update every 10 fs was commonly seen to increase the efficiency in protein simulations without leading to significant differences in thermodynamic and structural properties.^[^ ^15^, ^16^, ^17^ ^]^

Ideally, a force field should be independent of the simulation settings used at parameterization, but unfortunately using nonbonded interactions that are approximated by cutoff or lattice summation schemes, this is very hard if not impossible to achieve. Therefore, it is recommended to use simulation settings similar to the ones that were used upon parameterization. To parameterize the GROMOS force field a twin‐range, charge‐group‐based cutoff scheme, combined with a reaction‐field contribution was used. Recently, some discussion has come as to the validity of this approach.^[^ ^18^ ^]^ Recent studies indeed show that different results are obtained when using alternative cutoff schemes for, for example, the area per lipid,^[^ ^15^ ^]^ the radius of gyration of a dendrimer or constant pH simulations of membranes and proteins.^[^ ^16^ ^]^ Also, the thermodynamic properties of small molecules may be affected.^[^ ^19^ ^]^ We recently showed for small molecules, that these differences are not due to the use of the twin‐range, but may be attributed to the use of lattice sum electrostatics, or the switch to an atomistic rather than group‐based cutoff scheme.^[^ ^17^ ^]^ In this study, we aim to expand this analysis to a large number of simulations of proteins, such that statistically sound conclusions can be drawn with respect to any observed differences.

## 2 METHODOLOGY

The investigation is based on a set of 52 protein structures described by Setz et al.^[^ ^20^, ^21^ ^]^ This set consists of 39 structures obtained by X‐ray diffraction and 13 obtained by NMR experiments. Simulations were performed using the GROMOS11 software package and the GROMOS 54A8 force field.^[^ ^22^, ^23^ ^]^ The systems were solvated using the SPC water model and 0.15 M NaCl was added to the simulation box. For the equilibration, an eight‐step protocol was used. In the first six steps, the temperature was increased by 60 K at constant volume. At the same time, harmonic position restraints were loosened by one order of magnitude from an initial force constant of 2.5 × 10^4^ kJ mol^−1^ nm^−2^. Step 7 was used to instantiate the roto‐translational^[^ ^24^ ^]^ constraints on the solute atoms and in the last step pressure coupling was applied at 1 atm. The equilibration took 160 ps in total, 20 ps at every step.

Unless stated differently, the weak‐coupling scheme^[^ ^25^ ^]^ with relaxation times of 0.1 ps and 0.5 ps was used to keep the temperature and pressure constant at 298.15 K and 1 atm with an estimated isothermal compressibility of 4.575 × 10^−4^ (kJ mol^−1^ nm^−3^)^−1^. Solute and solvent were coupled to two separate temperature baths. The SHAKE^[^ ^26^ ^]^ algorithm was used with a relative tolerance of 10^−4^ to keep the bond lengths constrained to their minimum‐energy value, using a timestep of 2 fs. In this study, we compare four different sets of simulations, that differ in the way the nonbonded interactions are calculated. In the first set of simulations, the nonbonded interactions were calculated using a group‐based, twin‐range cutoff scheme (CG/TR), with a short‐range cutoff at 0.8 nm and a long‐range cutoff at 1.4 nm. The short‐range interactions were computed every timestep (2 fs) from a pairlist that was updated every 10 fs. The intermediate range interactions, up to the long‐range cutoff were computed at pairlist updates and kept constant in between. A reaction‐field contribution^[^ ^14^ ^]^ was added to all electrostatic interactions to account for a homogeneous medium beyond the long‐range cutoff with a relative dielectric constant of 61.^[^ ^27^ ^]^ In the second set of simulations, the frequency of the pairlist update and the calculation of intermediate‐range interactions were set to every 2 fs, resulting in a single‐range pairlist scheme (CG/SR). In the third set of simulations, the cutoff was applied based on interatomic distances (AT/TR). For the fourth set of simulations, the protein was treated atomistic, by treating every atom of the solute as a separate charge‐group, while the solvent was treated as in the charge‐group simulations (solute‐atomistic, SA/TR). Every protein system was simulated for all four cutoff schemes in triplicates for 15 ns, yielding in a total simulation time of around 10 μs.

For the simulations of pure SPC^[^ ^28^ ^]^ water, a box of 1,000 molecules was simulated in analogy to the protein simulations. The isothermal compressibility was estimated at 7.51 × 10^−4^ (kJ mol^−1^ nm^−3^)^−1^ and the relative dielectric constant of the reaction field was set to 78. The simulations were performed in triplicates for 10 ns each. Three different options for the cutoff were used, first an atomistic cutoff was used (AT), second a charge‐group‐based cutoff scheme was used with the center of the charge‐group being the center of geometry [CG(cog)] and in the third set of simulations the center of the charge‐group was placed on the oxygen atom of the water [CG(OW)]. These simulations were performed with the TR cutoff scheme.

The analysis was performed on the last 5 ns of the simulation trajectory. Structural features were compared using the RMSD_100_ proposed by Carugo and Pongor.^[^ ^29^ ^]^ Attempting to correct for differently sized proteins the RMSD_100_ normalizes the RMSD value to a protein of a 100 amino acid length. Hydrogen bond analysis was performed on the backbone of the protein. As geometric criterion, an acceptor–donor distance below 0.25 nm and an acceptor—hydrogen—donor angle larger than 120° was applied. The solvent accessible surface area of the protein was split, by amino acid type, in a nonpolar (A,C,F,I,L,M,V,W,Y) and a polar (remaining residues) contribution. The radius of gyration is calculated according to Equation (1) with *m*_*i*_ being the mass of atom i, *r*_*i*_ the position vector of every atom *i*, and *r*_*com*_ as the position vector of the center of mass of all atoms. *M* is the total mass of the protein.

(1) $$R_{\mathrm{gyr}}=\sqrt{\frac{1}{M}\sum_{i=1}^{N}m_{i}{\left(r_{i}-r_{\mathrm{com}}\right)}^{2}}$$

The occurrence of secondary structure motives was assigned using the Dictionary of Secondary Structures of Proteins, by Kabsch and Sander.^[^ ^30^ ^]^ For the structures resolved by NMR experiments, J‐coupling constants and NOE intensities were also evaluated for the statistical comparison of the protein set. J‐coupling constants were calculated via the related dihedral angle, using the empirical parameters for the Karplus relation proposed by Lindorff‐Larsen et al.^[^ ^31^ ^]^ Experimentally proposed NOE upper bounds for interproton distances were compared to simulated distance averages, computed as <*r* ^−3^>^−1/3^ and using pseudoatom‐corrections proposed by Wüthrich et al.^[^ ^32^ ^]^ The technical replicates of the simulation were pooled for this analysis. To investigate the structure of the solvent the radial distribution function (RDF) and the dipole–dipole orientation correlation function (DCF, *C*(*r*), Equation (2)) was used with $\hat{\mu_{i}}$ the direction of the water dipole moment.

(2) $$C\left(r\right)={〈\hat{\mu_{i}}\left(R\right)\hat{\mu_{j}}\left(R+r\right)〉}_{R}$$

To determine whether the variation of results obtained from different sets of simulations are significant, a mixed‐model linear analysis was used as described in Setz et al.^[^ ^20^, ^21^ ^]^ The *p*‐values of the binary contrasts of the different metrics were adjusted using the Benjamini–Yekutieli correction for multiple testing.^[^ ^33^ ^]^

Further investigations were conducted using the EGF domain of Spitz (PDB code: 3CA7). All different simulations were performed using six technical replicates and a simulation time of 15 ns. Apart from different cutoff schemes, a number of different reference temperatures were set for the temperature baths. Furthermore, two sets of simulations were conducted using a Nosé–Hoover chains thermostat with a chain length of 3. One set of simulations used particle–particle–particle‐mesh (P3M) lattice summation to account for long‐range electrostatics, using a real‐space cutoff of 0.8 nm and a grid spacing of 0.12 nm. The data are represented as mean values with *SD*s over the last 5 ns of the simulation. To compare the individual means, a pairwise *t* test was performed with a Holm–Bonferroni multiple testing correction.^[^ ^34^ ^]^

## 3 RESULTS AND DISCUSSION

Recent studies indicate that quantities obtained from molecular dynamics simulations depend on the treatment of the pairlist and the cutoff type.^[^ ^15^, ^16^, ^19^ ^]^ While there is some debate that observed differences are due to the use of a twin‐range cutoff scheme,^[^ ^18^ ^]^ this does not follow from the data in Table 1. No significant differences were observed for any of the analyzed properties between the CG/TR and CG/SR sets of simulations.

**TABLE 1:** Statistical analysis on the significance of differences. *p*‐Values obtained from a multivariate multilevel analysis on 52 proteins with 3 replicates each

	CG/TR vs. CG/SR		CG/TR vs. AT/TR	
Property	Significance	*p*‐Value	Significance	*p*‐Value
RMSD_100_	−	1	***	<.0001
No. H‐bond_*backbone*_	−	.3757	***	<.0001
SASA_*polar*_	−	1	***	<.0001
Radius of gyration	−	1	**	.0078
NOE violations^a^	−	1	*	.0151
*J*‐value^a^	−	1	−	.8488
SASA_*nonpolar*_	−	.14444	−	1
Occurrence of *α*‐helix	−	1	·	.0780
Occurrence of *π*‐helix	−	1	−	.5290
Occurrence of 3_10_‐helix	−	1	−	1
Occurrence of *β*‐strand	−	1	−	1
Occurrence of *β*‐bridge	−	1	−	1

Abbreviations: AT, atomistic; CG, charge‐group based; SR, single range; TR, twin range.

^a^ NMR data are available for a subset of 13 proteins.

**FIGURE 1:**
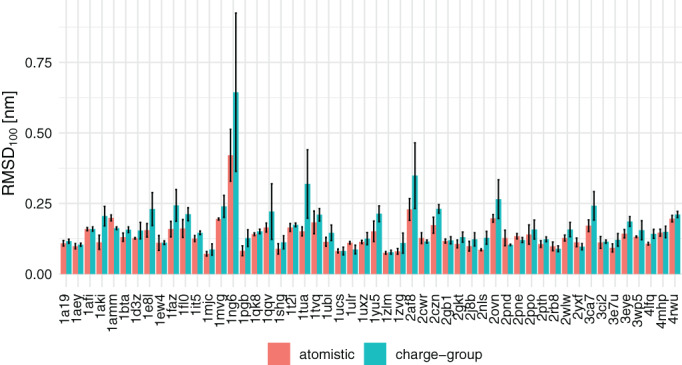
Average backbone RMSD_100_ values of all proteins simulated. The error bars indicate the *SD* from the mean of three replicates of one protein system [Color figure can be viewed at wileyonlinelibrary.com]

**FIGURE 2:**
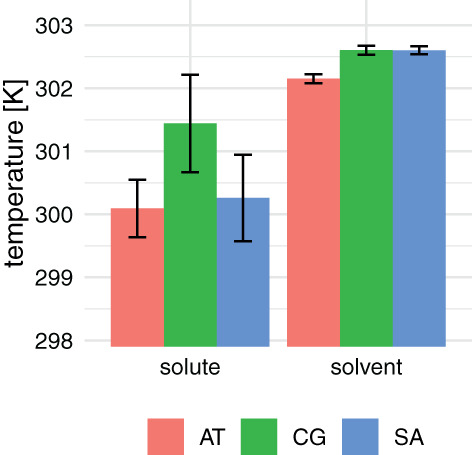
Average temperatures observed for solute and solvent degrees of freedom for atomistic (AT), charge‐group‐based (CG), and solute atomistic (SA) cutoff schemes. The reference temperature was set to 298.15 K. The error bars indicate the *SD* over all 156 simulations [Color figure can be viewed at wileyonlinelibrary.com]

On the other hand, significant differences are observed when comparing set CG/TR with set AT/TR for the RMSD_100_, the number of backbone hydrogen bonds, the solvent accessible surface area of polar amino acid residues, the radius of gyration, the violations of NOE distances, and the occurrence of *α*‐helical structures. Figure 1 shows the RMSD_100_ for all proteins. Simulations of 1ng6 show in general very high RMSD_100_ values. This structure of a cytosolic protein of unknown function consists of two 4‐helix bundles with a relatively flexible linker. Interestingly, the use of our recently updated parameter set 54A8_bb significantly reduced the values of RMSD_100_.^[^ ^3^ ^]^ For almost all proteins, the RMSD is higher in the case of the charge‐group‐based cutoff scheme. The differences in RMSD could be traced to the temperature of the solvent and solute in both simulation sets. The cutoff noise in either simulations leads to deviations from the target temperature. The solvent and solute temperatures were always lower in the simulations that used an atomistic cutoff than the ones that used a group‐based cutoff, as can be seen in Figure 2. The difference between the cutoff schemes was around 1.5 K for the solute degrees of freedom and only around 0.3 K for the solvent degrees of freedom. Although these differences are small, they seem to affect the system and lead to significant differences in the properties indicated in Table 1.

To determine if these differences are specific for soluble, structured proteins, we also performed the same set of simulations for the unstructured pentapeptide Ala_5_, see Figure S1 in the supplementary material. While the solute temperatures are generally maintained better for such a small peptide, the deviations from the reference values are still smaller for the atomistic cutoff scheme. For the solvent, the deviations from the reference temperature are similar to the values in Figure 2. The SASA values for atomistic cutoffs are in general lower than for charge‐group cutoffs and the radius of gyration and total number of backbone hydrogen bonds are very similar in all simulation settings.

**FIGURE 3:**
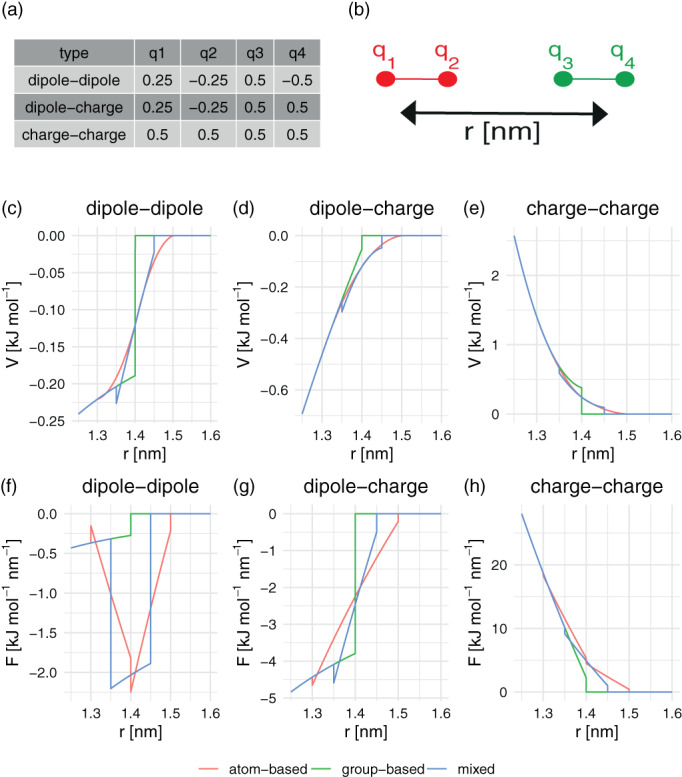
Simple one‐dimensional system (Panel (b)) to analyze the cutoff effect in detail. Two diatomic molecules are translated along the x‐axis and molecular interaction energies and forces are computed, for charges according to Panel (a). Interaction energy in the cutoff region for dipole–dipole (c), dipole–charge (d), charge–charge (e) system. The same for the forces in the lower panels (f)–(h) [Color figure can be viewed at wileyonlinelibrary.com]

To rationalize the differences we observed for the proteins, a simple one‐dimensional system as described in Figure 3 was used to investigate the energies and forces at the cutoff. Two diatomic molecules with different charge distributions were placed at different intermolecular distances. Both molecules were 0.1 nm in size. The electrostatic interaction and force along the molecular axes were calculated using different cutoff schemes. The “mixed” cutoff scheme is a combination of the atomistic and charge‐group‐based schemes, where charges q1 and q2 are treated as atomistic and charges q3 and q4 as charge group. The interaction energy between two atoms *i* and *j* is calculated using:

(3) $$V_{\mathrm{ij}}^{\mathrm{el}}=\frac{q_{i}q_{j}}{4\pi {ɛ}_{0}}\left[\frac{1}{r_{\mathrm{ij}}}-\frac{\frac{1}{2}C_{\mathrm{rf}}r_{\mathrm{ij}}^{2}}{R_{\mathrm{rf}}^{3}}-\frac{1-\frac{1}{2}C_{\mathrm{rf}}}{R_{\mathrm{rf}}}\right]$$

where *r*_*ij*_ is the interatomic distance, *C*_*rf*_ is a reaction‐field constant depending on the reaction‐field dielectric constant, and *R*_*rf*_ is the reaction‐field cutoff distance.^[^ ^14^ ^]^ The last distance‐independent term, ensures that the electrostatic energy approaches zero when *r*_*ij*_ = *R*_*rf*_.

In Figure 3c–e, the energies occurring around the 1.4 nm cutoff were plotted, for dipole–dipole, dipole–charge and charge–charge interactions. This example shows that for an atomistic cutoff scheme, the overall energy goes to zero more smoothly than for charge‐group‐based cutoffs. This can be explained from Equation (3), which goes to zero if the interatomic distance *r*_*ij*_ = *R*_*rf*_. However, in the group‐based cutoff scheme, some atoms may no longer interact at distances shorter than *R*_*rf*_, or still interact beyond this distance, leading to sudden jumps in the electrostatic interaction energy between the molecules. These sudden changes lead to larger cutoff noise, and hence demand more heat exchange with the temperature baths to maintain the temperature at the target value. Indeed, in previous work, we observed that the difference between AT and CG becomes smaller when using larger cutoffs, as the size of the energy jumps diminishes.^[^ ^16^ ^]^ For the forces in Figure 3f–h, however, the dipole–dipole interaction leads to irregular spikes around the cutoff for the atomistic cutoff scheme. At distances where some atoms no longer interact, the molecular interaction changes to a dipole–charge or charge–charge interaction, with different slopes in the energy profile, and hence different forces. As the two molecules move further apart, the forces fluctuate strongly. The blue line of the mixed‐cutoff scheme approximates the smooth energy profile of the atomistic cutoff scheme, and also shows the artificial spikes in the dipole–dipole forces.

**FIGURE 4:**
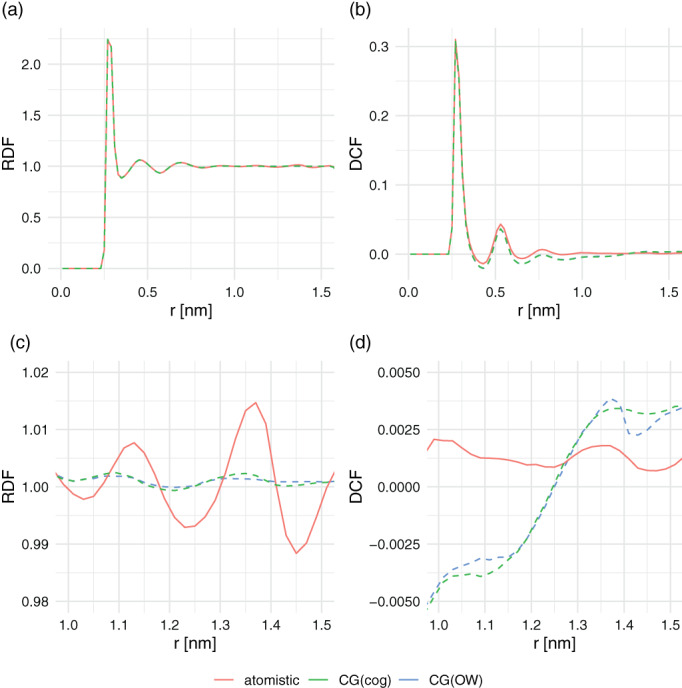
Radial distribution function of water oxygen atoms (a) and dipole correlations function for water (b). Panels (c) and (d) zoom in to the region around the cutoff (1.4 nm) [Color figure can be viewed at wileyonlinelibrary.com]

The effect of the irregular forces in the atomistic cutoff scheme around the cutoff can be seen by analyzing the RDFs and DCFs for a box of 1,000 SPC water molecules (Figure 4). The close‐up of the RDF shows an artificial structure around the cutoff region for the simulations using an atomistic cutoff scheme. For the DCF, a slight anticorrelation can be observed for the charge‐group case, as was observed previously.^[^ ^12^, ^16^, ^17^ ^]^ Different centers of the charge‐group do not seem to have a major influence on the RDF and the DCF (compare CG(cog) and CG(OW)). To ensure that this observation is not a peculiarity of the SPC water model, we have performed AT and CG(OW) simulations of the TIP4P water model, and find very similar artifacts around the cutoff (Figure S2 in supplementary material).

Following up on the mixed cutoff scheme in Figure 3, the 52 proteins were simulated using a cutoff scheme in which the protein atoms were treated as individual groups, while the solvent was treated using a group‐based cutoff [CG(OW)]. Table 2 shows the differences in the monitored protein quantities. It can be observed that the solute atomistic cutoff set leads to the proteins behaving comparably to the atomistic case, except for the SASA_*polar*_ which seems to be governed by the water being treated as charge‐group. This is in agreement with the observations in Figure 3, where the mixed cutoff scheme is most similar to the atomistic scheme. Also for the temperatures in Figure 2, the solute behaves similar to the atomic case and the solvent similar to the charge‐group case.

**TABLE 2:** Statistical analysis on the significance of differences. *p*‐Values obtained from a multivariate multilevel analysis on 52 proteins with 3 replicates each

	AT/TR vs. SA/TR		CG/TR vs. SA/TR	
Property	Significance	*p*‐Value	Significance	*p*‐Value
RMSD_100_	−	1	***	<.0001
No. H‐bond_*backbone*_	−	.1382	**	.0016
SASA_*polar*_	***	<.0001	−	1
Radius of gyration	−	1	−	.1382
NOE violations^a^	−	1	·	.0551
*J*‐value^a^	−	1	−	1
SASA_*nonpolar*_	−	.5494	−	.2303
Occurrence of *α*‐helix	−	1	**	.0075
Occurrence of *π*‐helix	−	1	−	1
Occurrence of 3_10_‐helix	−	1	−	.8488
Occurrence of *β*‐strand	−	1	−	1
Occurrence of *β*‐bridge	−	1	−	1

Abbreviations: AT, atomistic; CG, charge‐group based; SA, solute‐atomistic; TR, twin range.

^a^ NMR data are available for a subset of 13 proteins.

**FIGURE 5:**
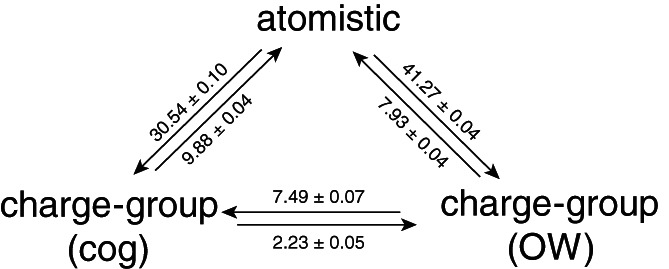
Change in nonbonded electrostatic energy in kJ mol^−1^ upon reanalysis of trajectories of pure water simulations using an atomistic cutoff (AT), a charge‐group‐based cutoff using the center of geometry CG(cog) and using a charge‐group‐based cutoff at the oxygen of the water molecule CG(OW)

Next, we turn our attention to the energetic differences between the different cutoff schemes. The potential energy was recalculated for the configurations that were obtained from simulations with one cutoff scheme, applying an alternative cutoff scheme. Figure 5 shows the resulting change in potential energy for the simulations of pure water. All values in this figure are positive, which follows from the fact that configurations are generated that are most favorable for the cutoff scheme used in the simulation. However, there is an asymmetry in the values. As can be seen in this picture, the difference in energy going from an atomistic simulation to a charge‐group‐based cutoff scheme is much more unfavorable than vice versa. This is because in the atomistic case, a higher water density is artificially observed before the cutoff. Furthermore, water molecules at the cutoff will orient themselves such that unfavorable interactions are placed out of the cutoff. Reintroducing these in a group‐based recalculation subsequently leads to unfavorable interactions. This is in line with the differences in density and orientations at the cutoff as seen in Figure 4. Similarly, the much smaller differences between the CG(cog) and CG(OW) may be explained by the difference between the green and blue curves of the DCF at exactly 1.4 nm. Using the oxygen atom as the center of the water molecules, leads to a slightly larger positive correlation just before the cutoff, followed by a slight drop in the correlation beyond the cutoff.

**FIGURE 6:**
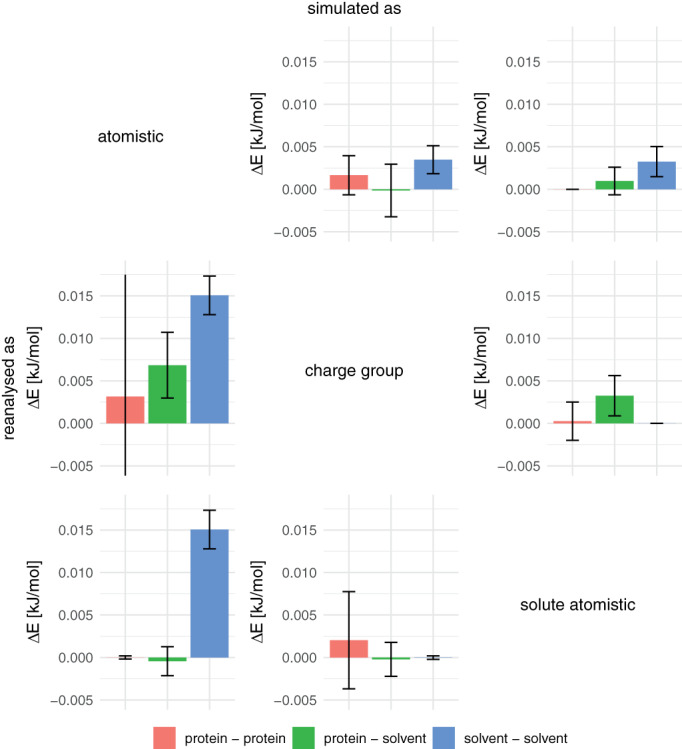
Differences in the nonbonded electrostatic energy upon reanalysis of protein trajectories. The protein–protein and solvent–solvent energies were normalized by the number of atoms in the respective sets ($N_{a}^{\text{solute}}$ and $N_{a}^{\text{solvent}}$, respectively) and the protein‐solvent interactions were normalized by ($N_{a}^{\text{solute}}\cdot N_{a}^{\text{solvent}}$)${}^{\frac{1}{2}}$ [Color figure can be viewed at wileyonlinelibrary.com]

A similar recalculation of the potential energy was performed for the protein simulations. Figure 6 shows the change in energy from all three different cutoff schemes reanalyzed using the other schemes, separated into protein–protein, protein–solvent, and solvent–solvent contributions. All contributions were normalized with respect to the number of atoms prior to averaging over the proteins. Again, the difference in energy seems always unfavorable, but statistical significance is only reached for few energy terms and simulation settings. The most pronounced difference in terms of energy is seen in the solvent–solvent interactions when recalculating a simulation that was performed with an atomistic cutoff to a (solvent) group‐based cutoff. This is in line with the larger values for similar changes in Figure 5. We interpret this such, that the added structure in the solvent that is observed in the RDFs for atomistic cutoff simulations is also relevant in the protein simulations and should be avoided.

**FIGURE 7:**
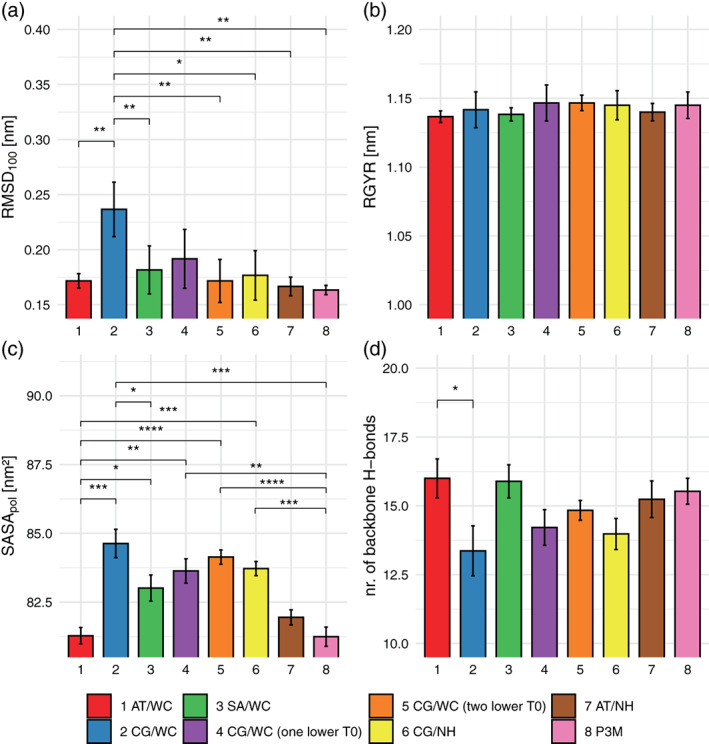
Simulations of Spitz EGF using seven different temperature settings (Table 3), with average RMSD_100_ in Panel (a), the radius of gyration in Panel (b), the SASA_*pol*_ in Panel (c), and the number of backbone hydrogen bonds in Panel (d) [Color figure can be viewed at wileyonlinelibrary.com]

To test if the changes that are observed between atomistic and group‐based cutoff schemes can be compensated by different settings of the temperature baths, the EGF domain of Spitz (PDB‐Id: 3ca7) was simulated using eight different simulation settings. The settings are outlined in Table 3 and include the use of lower reference temperatures, the use of a Nosé–Hoover chains thermostat and the use of P3M for the long‐range electrostatics. Every individual parameter set was simulated in sixtuples. The actual average temperatures observed in the simulations are also listed in this table. The P3M simulations show, that a complete removal of the cutoff noise, reduces the solute temperature close to the target, while the solvent temperature remains high. This suggests that the noise in the solvent is mainly due to another source, possibly related to the use of distance constraints. Figure 7 shows the effects for the properties for which significant differences were observed in Table 1. For the RMSD_100_, significant differences can be seen comparing the charge‐group‐based cutoff to almost every other simulation setting. This confirms that a more exact temperature control can indeed reduce the RMSD_100_ values. For the radius of gyration, there were no significant differences observed and for the number of hydrogen bonds the differences are only between the atomistic and charge‐group‐based cutoff schemes. For SASA_*pol*_, significant differences can be seen between the atomistic and all other simulated sets, except for the atomistic simulations performed using a Nosé–Hoover thermostat and the P3M simulations.

**TABLE 3:** Simulation settings for the additional sets of simulations of the EGF protein. Cutoff schemes used are AT, CG, SA, or P3M. Thermostats refer to WC or NH chains. In simulation Sets 4 and 5, the reference temperatures were reduced to obtain observed temperatures closer to the target (Set 4) or to the AT setup (Set 5)

No.	Cutoff	Thermostat	Reference solute temperature (K)	Observed solute temperature (K)	Reference solvent temperature (K)	Observed solvent temperature (K)
1	AT	WC	298.15	299.42	298.15	302.27
2	CG	WC	298.15	301.75	298.15	302.63
3	SA	WC	298.15	299.76	298.15	302.69
4	CG	WC	295.85	299.28	295.85	300.33
5	CG	WC	296.02	299.79	297.75	302.27
6	CG	NH	298.15	299.01	298.15	300.28
7	AT	NH	298.15	298.34	298.15	300.13
8	P3M	NH	298.15	299.39	298.15	302.23

Abbreviations: AT, atomistic; CG, charge‐group based; NH, Nosé–Hoover; P3M, particle–particle–particle mesh; SA, solute‐atomistic; WC, weak‐coupling.

**FIGURE 8:**
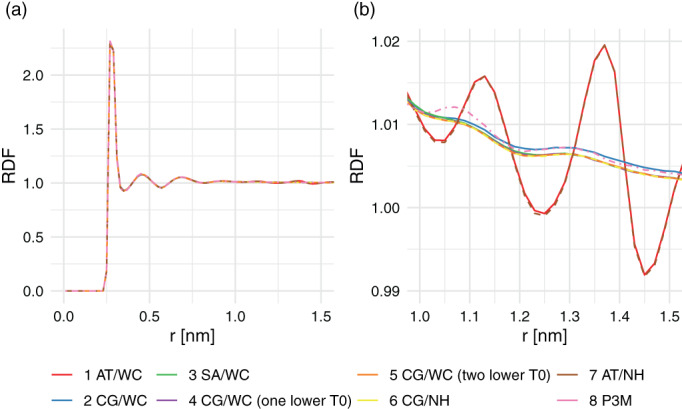
Radial distribution function of the solvent obtained in the simulations of Spitz EGF in Panel (a) and the zoomed representation in Panel (b). The dashed lines represent simulations where a Nosé–Hoover thermostat was used [Color figure can be viewed at wileyonlinelibrary.com]

Figure 8 shows the water–water RDF in the Spitz simulations. The overall downward trend in Panel (b) can be explained from the fact that the protein occupies a considerable volume in the simulation box. The curves for the charge‐group and atomistic cutoff schemes and temperature settings can be clearly distinguished. As expected, the artificial structure at the cutoff for AT simulations persists in the protein simulation. The effect of a more precise temperature control is minor, with the blue curve (CG/TR) slightly above the other CG curves. The P3M curve shows slightly more structure around 1.1 nm, but otherwise is most similar to the CG schemes. The SA scheme is indistinguishable from the CG schemes with a more precise solute temperature, in spite of the higher solvent temperature (Table 3). These data suggest that a close look at the temperature control of simulations remains an important check for any biomolecular simulation.

## 4 CONCLUSIONS

We described simulations of 52 protein systems, using four different cutoff and pairlist schemes. No significant differences were observed for any of the analyzed properties when comparing the twin‐range cutoff scheme to a single‐range cutoff scheme. However, the choice of the entities to which the cutoff is applied (atomistic vs. group‐based) does have a significant influence on some of the molecular properties. Investigations on pure water simulations show that using an atomistic cutoff leads to artificial structure in the water at the cutoff, whereas the water‐dipoles seem to be slightly anticorrelated in the charge‐group case. Re‐analysis of simulations with alternative cutoff schemes suggests, that these structural effects also propagate into the energetics of the solvent in protein‐water simulations. Simulations of the Spitz EGF protein suggest that proper control of the effective simulation temperature can remove the observed differences in the analyzed properties. A solute‐atomistic simulation scheme seems to have the same effect, leading to less noise in the protein degrees of freedom, while still avoiding the artificial structure of the solvent at the cutoff. This approach has the added advantage that the speed‐up of using group‐based water molecules can be maintained. Overall, we conclude that while the cutoff noise may be less with an atomistic cutoff, due to smoother energy curves, this comes as the expense of artificial structure in the solvent, due to irregular forces at the cutoff. A solute‐atomistic cutoff scheme or simply a close look at the settings of the temperature baths is sufficient to control the charge‐group‐based cutoff noise.

## Supporting information


**Appendix S1**: Supporting Information
