# Supplementary material for: The effect of different cutoff schemes in molecular simulations of proteins
Source: J Comput Chem. 2020 Oct 7;41(32):2740–9. doi: 10.1002/jcc.26426 (PMC7756334; doi:10.1002/jcc.26426)
Supplement: Supplementary file 1 — Appendix S1: Supporting Information [file JCC-41-2740-s001.pdf]

Supplementary Material

The effect of different cutoff schemes in molecular simulations of proteins

Diem Matthias, Oostenbrink Chris *

September 6, 2020

*Institute for Molecular Modeling and Simulation, University of Natural Resources and Life Sciences, Muthgasse 18, 1190 Vienna, Austria

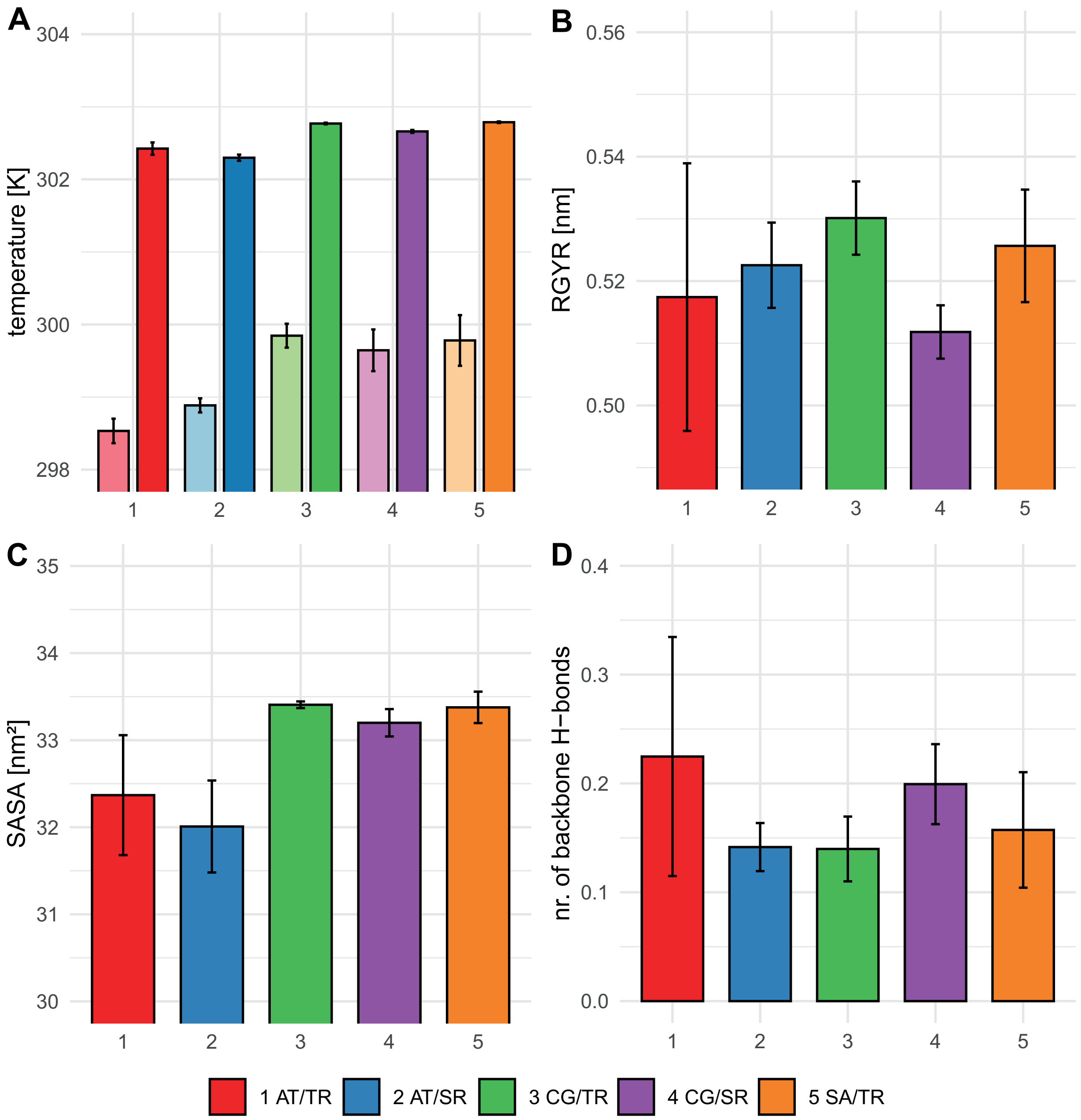

Figure S1: Simulations of ALA_5 using five different cutoff settings, with average temperature of the solute (light colours) and solvent (dark colours) in panel A, the radius of gyration in panel B, the solvent accessible surface area (SASA) in panel C and the number of backbone hydrogen bonds in panel D.

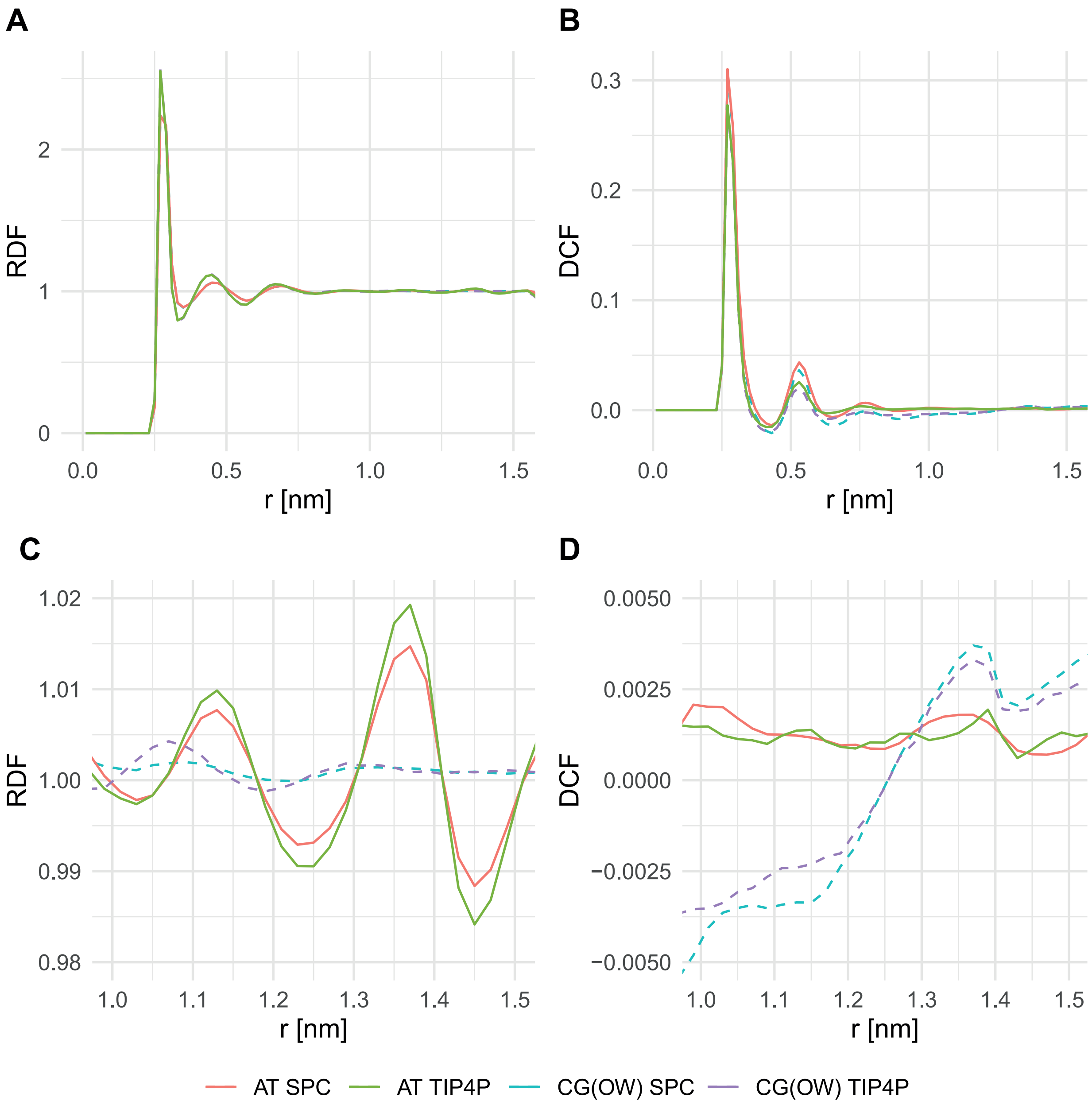

Figure S2: Radial distribution function of water oxygen atoms (A) and dipole correlations function for water (B), comparing SPC and TIP4P water models. Panels (C) and (D) zoom in to the region around the cutoff (1.4 nm).
